# Determination of Surface Accessibility of the Cellulose Substrate According to Enzyme Sorption

**DOI:** 10.3390/polym11071201

**Published:** 2019-07-18

**Authors:** Ekaterina M. Podgorbunskikh, Aleksey L. Bychkov, Oleg I. Lomovsky

**Affiliations:** 1 Institute of Solid State Chemistry and Mechanochemistry, Siberian Branch, Russian Academy of Sciences, ul. Kutateladze 18, Novosibirsk 630128, Russia; 2 Novosibirsk State Technical University, pr. K. Marksa 20, Novosibirsk 630073, Russia

**Keywords:** cellulose, reactivity, surface area, enzymes, sorption capacity, mechanical activation, reaction rate, interface

## Abstract

As a heterogeneous process, enzymatic hydrolysis depends on the contact area between enzymes and the cellulose substrate. The surface area of a substrate is typically evaluated through the sorption of gases (nitrogen, argon, or water vapor) or sorption of high-molecular-weight pigments or proteins. However, lignocellulosic biomass uninvolved in the reaction because of inefficient binding or even the complete inhibition of the enzymes on the surface consisting of lignin or inorganic compounds is erroneously taken into account under these conditions. The initial rate of enzymatic hydrolysis will directly depend on the number of enzymes efficiently sorbed onto cellulose. In this study, the sorption of cellulolytic enzymes was used to evaluate the surface accessibility of the cellulose substrate and its changes during mechanical pretreatment. It was demonstrated that for pure cellulose, mechanical activation did not alter the chemical composition of the surface and the initial rate of hydrolysis increased, which was inconsistent with the data on the thermal desorption of nitrogen. New active cellulose sorption sites were shown to be formed upon. the mechanical activation of plant biomass (wheat straw), and the ultimate initial rate of hydrolysis corresponding to saturation of the accessible surface area with enzyme molecules was determined.

## 1. Introduction

The biorefinery of plant biomass to useful products starts with a heterogeneous interaction between substrates and reagents (acids, alkalis, or enzymatic complexes). From the perspective of the kinetics of heterogeneous processes, the substrate surface area directly affects the initial reaction rate [1]. The specific surface area, the most frequently used parameter, depends not only on particle size, but also on the parameters of pores and cracks. However, there are a number of problems that make it impossible to properly evaluate the substrate surface area available for interaction with the reagents. Whereas the effect of porosity can often be considered negligible for native biomass, its morphology changes dramatically after pretreatment aimed at enhancing the reactivity of the biomass [2]. Pretreatment (e.g., mechanical activation) significantly alters surface properties: it reduces grain size, results in formation of cracks and pores and component redistribution in the near-surface layer [3,4,5].

Thermal gas desorption using the Brunauer–Emmet–Teller equation (the BET method) is the most common technique utilized to study the surface properties [6]. A number of assumptions are made in this method. It is assumed that a solid surface is energetically homogeneous (the adsorption sites are identical), and the “horizontal” interactions between molecules within the layer plane are not taken into account. It is worth mentioning that lignocellulosic biomass is a macroporous material, and so a measurement uncertainty arises [1].

Attempts have been made to either refine and modify the conventional BET equation [7,8] or modify the gas desorption methods. Thus, Gregg and Sing [9] suggested using an approximation to measure the surface area with respect to a certain reference sample, usually of inorganic nature.

**Figure 1:**
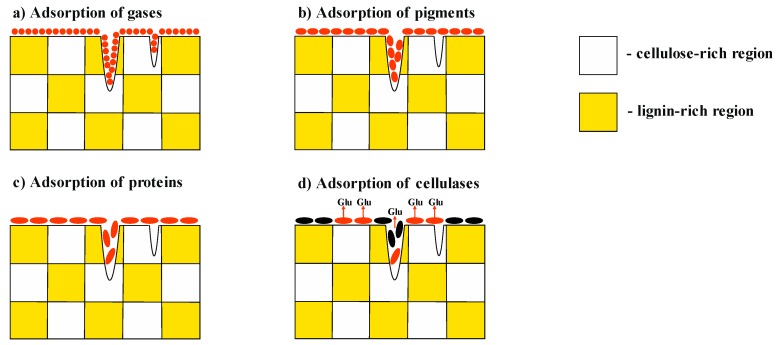
Scheme of sorption of low-molecular-weight gases (**a**), pigments (**b**), proteins (**c**), and cellulases (**d**) onto the surface of lignocellulosic biomass.

However, even with these modifications, the gas desorption methods cannot properly measure the surface area accessible for enzymes (Figure 1). In particular, the size of the catalytic core of endoglucanase from *Trichoderma reesei* is ~4–5 nm, making the surface of smaller pores inaccessible [10].

The adsorption of pigments [11] or high-molecular-weight substances (proteins) [12] is a more accurate method for determining the accessible surface area of the substrate. Hence, Hong et al. [13] studied the sorption of non-hydrolytic recombinant protein carrying a cellulose-binding module and green fluorescent protein (which is similar to endoglucanase I from *Trichoderma reesei*) onto cellulose specimens.

However, even the sorption of proteins and pigments cannot show onto which surface the test compound has been sorbed. The surface of real-life objects consists not only of cellulose but also of polyphenols, inorganic components, and waxes. The adsorption of an enzyme onto a surface comprised of polyphenols or waxes leads to inefficient binding or even the complete inhibition of the enzyme [2,5]. The initial rate of enzymatic hydrolysis will be directly dependent on the number of enzymes sorbed onto reactive cellulose. It should be mentioned, however, that in our manuscript the term “reactive cellulose” implies not just cellulose whose surface is accessible for enzymes; reactive cellulose must also have a disordered (amorphous) crystalline structure whose hydrolysis rate is dramatically higher than that of crystallites.

Therefore, this study aimed to determine the accessibility of the cellulose substrate surface and changes in it occurring during mechanical treatment.

## 2. Materials and Methods

The reagents used in this study were as follows: sodium azide (SIGMA-Aldrich, Moscow, Russian Federation), sodium acetate (SIGMA-Aldrich, Moscow, Russian Federation), bidistilled water, potassium ferricyanide (III) (SIGMA-Aldrich, Moscow, Russian Federation), sodium hydroxide (SIGMA-Aldrich, Moscow, Russian Federation), D(+)-glucose (99%, SIGMA-Aldrich, St. Quentin Fallavier, France), acetic acid (SIGMA-Aldrich, St. Luis, MI, USA), and CelloLux-A enzymatic complex (Sibbiopharm Ltd., Berdsk, Russia).

α-Cellulose (SIGMA-Aldrich, catalog number C8002) and wheat straw (*Triticum durum L.*) were used as study objects. Wheat straw is the conventional lignocellulosic plant biomass with a well-described structure and is commonly utilized as a model study object [14]. Wheat straw used in this study was harvested in September 2017 in the Novosibirsk region (Russia). The wheat straw contained 49.2 ± 0.2% cellulose (evaluated using the Kushner’s method), 21.5 ± 0.2% hemicellulose, 20.3 ± 0.3% acid-insoluble lignin, 7.4 ± 0.5 extractive substances, and 3.0 ± 0.1% ash.

Enzymatic hydrolysis of cellulosic and lignocellulosic substrates was performed at 50 °C in an acetate buffer (pH 4.7) with the 1:40 hydromodule; the concentration of the enzyme complex was gradually increased. The initial reaction rate was determined after the enzymatic hydrolysis had been conducted for 30 min using the Hagedorn–Jensen ferrocyanide method according to the content of reducing saccharides. Carbonyl groups of saccharides were reduced with 0.06% solution of potassium hexacyanoferrate in an alkaline medium. Concentrations of the reducing saccharides were calculated using a calibration curve; D(+)-glucose solutions were used as a reference at λ = 420 nm with respect to water.

The degree of cellulose crystallinity was measured by powder X-ray diffraction analysis on a D8 Advance diffractometer (Bruker, Karlsruhe, Germany) and calculated using the Segal, Rietveld, and Lorenz methods [15,16]. Granulometric analysis was conducted using an Analysette-3 Pro vibratory sieve shaker equipped with a set of sieves (mesh size, 20–1000 µm) (FRITSCH, Markt Einersheim, Germany) and a Microsizer 201 laser particle size analyzer equipped with an ultrasonic disperser (VA Instalt, Saint Petersburg, Russia). The specific surface area of the samples was determined according to thermal desorption of nitrogen on a Sorptometer M instrument (Catakon, Novosibirsk, Russia) using the BET method [6].

Mechanical pretreatment of individual α-cellulose and wheat straw was conducted on an AGO-2 planetary ball mill equipped with a water-cooling system; the rotational speed of the reactors was 630 rpm, and the acceleration of the grinding bodies was 200 m/s^2^. The weight of the grinding bodies (steel balls 5 mm in diameter) was 200 g, the weight of the biomass being mechanically activated was 10 g, and the pretreatment duration was varied between 2 and 30 min.

## 3. Results and Discussion

The accessible surface area is the key parameter responsible for the yield at the initial stage of enzymatic hydrolysis. There is a direct correlation between glucose yield and the accessible surface area at hydrolysis durations up to 2 h, which makes it possible to identify the pattern of changes on the surface of plant biomass according to the initial rate of enzymatic hydrolysis.

**Figure 2:**
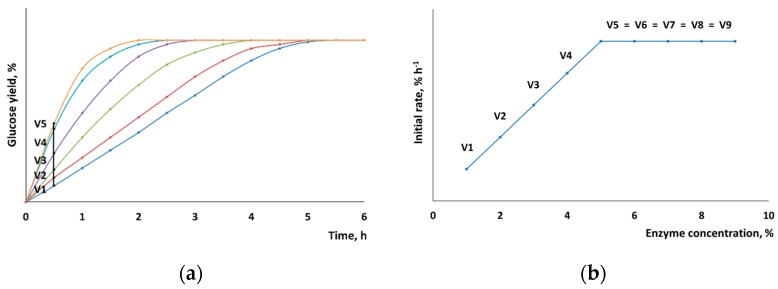
Determination of the maximum initial rate of hydrolysis by hypothetical kinetic curves: (**a**) a set of hypothetical kinetic curves with different substrate–enzyme ratios; (**b**) the initial rate of hydrolysis as a function of enzyme concentration.

In order to determine its reaction rate, enzymatic hydrolysis was conducted for 30 min, with the yield of low-molecular-weight carbohydrates being controlled. The reaction time was selected for a linear section of the kinetic curve of enzymatic hydrolysis, where the slope of the curve remained unchanged and was proportional to the percentage of the enzymes that had reacted with the cellulose surface. When planning the experiment design, we proceeded from the hypothesis that if enzyme concentration is gradually increased, one can find a point at which the reaction rate will no longer increase because the entire surface is occupied by the enzymes. Figure 2 shows a hypothetical example illustrating the principle used to determine the maximum reaction rate of hydrolysis.

**Figure 3:**
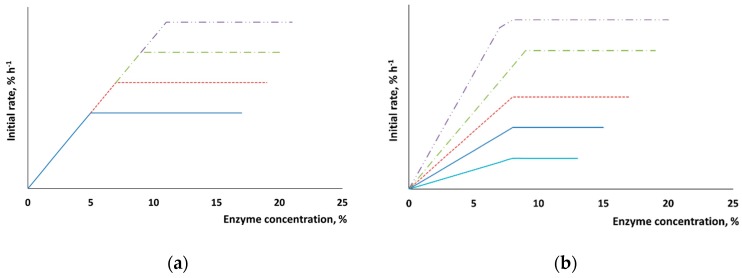
The putative changes in the initial rate of hydrolysis after pretreatment of cellulose (**a**) and the lignocellulosic biomass (**b**).

When the chemical composition of the surface remains unchanged (e.g., for pure cellulose), the maximum rate of hydrolysis after pretreatment is expected to increase in proportion to the newly formed surface (Figure 3a). The interaction between enzymes and native lignocellulosic biomass is hindered by the presence of lignin and hemicelluloses on the surface, and this fact is supposed to somehow affect the sorption properties (Figure 3b). In addition to changes in the maximum rate of hydrolysis, one can expect that the dependence between the surface area of the added enzymes and the initial rate of hydrolysis will also be altered.

**Figure 4:**
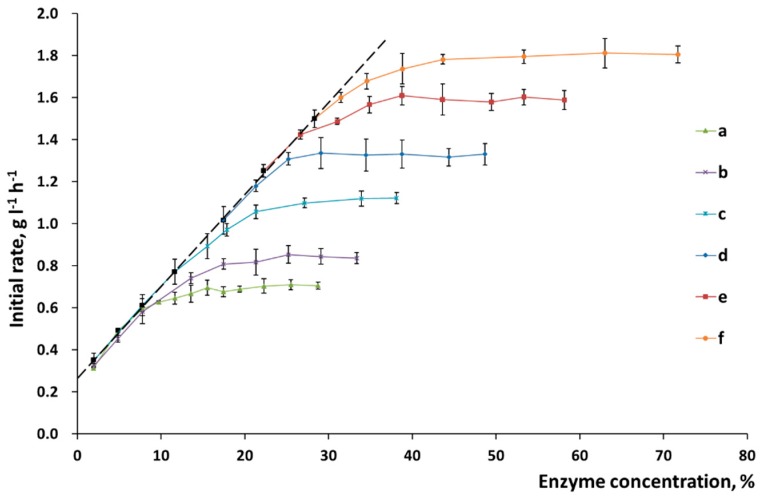
The initial rate of hydrolysis of α-cellulose after mechanical activation: (**a**) without activation and after activation for (**b**) 2 min; (**c**) 5 min; (**d**) 10 min; (**e**) 20 min; and (**f**) 30 min.

These assumptions were verified experimentally. It was shown in Figure 4 that when the substrate surface consisted of pure cellulose, the position of the point corresponding to the maximum rate of hydrolysis shifted linearly depending on the time of mechanical activation.

Mechanical activation makes additional surface accessible as the biomass becomes disintegrated and the crystalline regions of cellulose are amorphized. In our experiment, the maximum reactivity of α-cellulose was not reached even after the mechanical pretreatment for 30 min. Although the maximum possible disintegration was reached more quickly and amorphization took place, as shown in Table 1, the capacity of the cellulose substrate with respect to the enzymes further increased. The accessible surface area continued to increase with pretreatment time, but no changes in the specific surface area measured using the BET method, or the degree of crystallinity, or particle size were observed. The reason behind this fact is that a deeper disordering of the supramolecular structure of X-ray amorphous cellulose takes place at long pretreatment times, which cannot be detected by X-ray diffraction analysis.

**Figure 5:**
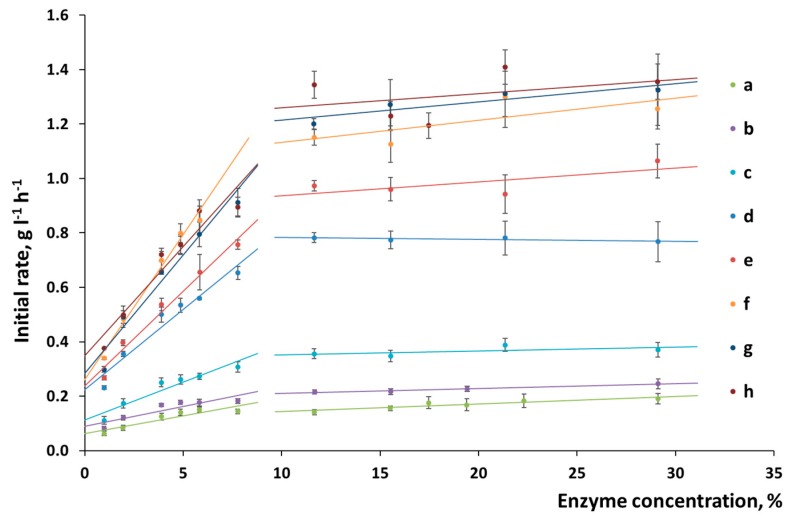
The initial rate of hydrolysis of wheat straw as a function of enzyme concentration: (**a**) without mechanical activation and after mechanical activation for (**b**) 2 min; (**c**) 5 min; (**d**) 10 min; (**e**) 15 min; (**f**) 20 min; (**g**) 25 min; and (**h**) 30 min.

The chemical composition of the surface of plant biomass is more complex than that of the substrates made of pure cellulose. Changes in the slope of the straight line in the “reaction rate versus enzyme concentration” coordinates make it possible to determine the features of the surface formed during pretreatment (Figure 4). The slope of the straight line for α-cellulose was not changed, thus directly indicating that the chemical composition of the surface was homogeneous and constant (Figure 4). In the case in which wheat straw was pretreated, the changed slope demonstrates that the nature of enzyme sorption onto the substrate surface was altered. Mechanical activation increases the slope (Figure 5), thus indicating that the percentage of active sorption sites on the cellulose surface has also risen.

Having analyzed the changes in the initial rate of hydrolysis (Figure 5), one can see that ultimate initial rate corresponding to the point of substrate saturation with enzymes (after mechanical activation for 20–30 min) was reached for the lignocellulosic biomass. Treatment duration does not increase the initial hydrolysis rate any further. The difference between the observed processes can be attributed to the presence of lignin, which prevents the separation of cellulose fibers in native biomass. This fact demonstrates that optimal conditions of mechanical treatment should be selected, which would allow one to liberate as much accessible surface as possible so that the complex of cellulolytic enzymes could be sorbed onto it.

## 4. Conclusions

The sorption of cellulases onto the substrate surface provides data on the surface area accessible for enzymes, which is not taken into account when determining the surface area by the sorption of low-molecular-weight substances. For pure α-cellulose, mechanical activation without altering the chemical composition of the surface enhances cellulose accessibility for the enzymes. Changes in the supramolecular structure of cellulose taking place upon longer mechanical activation are so deep that the initial rate of hydrolysis increases even after the maximum possible disintegration had been reached.

When the substrate surface is hindered by the presence of the lignocarbohydrate matrix, mechanical activation modifies the chemical composition of the surface as demonstrated by the increased slope of the straight lines responsible for the features of enzyme sorption onto a substrate. The maximum rate of hydrolysis corresponding to the ultimate saturation of the accessible surface area is reached after mechanical activation for 20 min.

## Figures and Tables

**Table 1:** Changes in the specific surface area, degree of crystallinity and particle size of α-cellulose and wheat straw.

Activation Time, min	α-Cellulose			Wheat Straw		
	Specific Surface Area, m^2^/g	Degree of Crystallinity, %	Average Particle Size, µm	Specific Surface Area, m^2^/g	Degree of Crystallinity, %	Average Particle Size, µm
0 (initial)	1.6 ± 0.2	71 ± 2	75 ± 3	2.2 ± 0.2	67 ± 2	252 ± 14
2	1.4 ± 0.1	55 ± 3	79 ± 5	1.9 ± 0.2	64 ± 3	173 ± 11
5	2.0 ± 0.2	30 ± 4	62 ± 3	2.4 ± 0.2	53 ± 4	47 ± 4
10	2.4 ± 0.2	22 ± 5	35 ± 3	4.3 ± 0.4	26 ± 4	13 ± 2
20	3.0 ± 0.3	11 ± 1	26 ± 2	4.5 ± 0.5	20 ± 2	20 ± 2
30	2.8 ± 0.3	12 ± 4	22 ± 2	3.4 ± 0.3	11 ± 5	23 ± 2
